# Consequences of reprogramming acetyl-CoA metabolism by 2,3,7,8-tetrachlorodibenzo-*p*-dioxin in the mouse liver

**DOI:** 10.1038/s41598-023-31087-9

**Published:** 2023-03-13

**Authors:** Giovan N. Cholico, Karina Orlowska, Russell R. Fling, Warren J. Sink, Nicholas A. Zacharewski, Kelly A. Fader, Rance Nault, Tim Zacharewski

**Affiliations:** 1 Biochemistry and Molecular Biology, Michigan State University, Biochemistry Building, 603 Wilson Road, East Lansing, MI 48824 USA; 2 Institute for Integrative Toxicology, Michigan State University, East Lansing, MI 48824 USA; 3 Microbiology & Molecular Genetics, Michigan State University, East Lansing, MI 48824 USA

**Keywords:** Autophagy, Mechanisms of disease, Transcriptomics

## Abstract

2,3,7,8-Tetrachlorodibenzo-*p*-dioxin (TCDD) is a persistent environmental contaminant that induces the progression of steatosis to steatohepatitis with fibrosis in mice. Furthermore, TCDD reprograms hepatic metabolism by redirecting glycolytic intermediates while inhibiting lipid metabolism. Here, we examined the effect of TCDD on hepatic acetyl-coenzyme A (acetyl-CoA) and β-hydroxybutyrate levels as well as protein acetylation and β-hydroxybutyrylation. Acetyl-CoA is not only a central metabolite in multiple anabolic and catabolic pathways, but also a substrate used for posttranslational modification of proteins and a surrogate indicator of cellular energy status. Targeted metabolomic analysis revealed a dose-dependent decrease in hepatic acetyl-CoA levels coincident with the phosphorylation of pyruvate dehydrogenase (E1), and the induction of pyruvate dehydrogenase kinase 4 and pyruvate dehydrogenase phosphatase, while repressing ATP citrate lyase and short-chain acyl-CoA synthetase gene expression. In addition, TCDD dose-dependently reduced the levels of hepatic β-hydroxybutyrate and repressed ketone body biosynthesis gene expression. Moreover, levels of total hepatic protein acetylation and β-hydroxybutyrylation were reduced. AMPK phosphorylation was induced consistent with acetyl-CoA serving as a cellular energy status surrogate, yet subsequent targets associated with re-establishing energy homeostasis were not activated. Collectively, TCDD reduced hepatic acetyl-CoA and β-hydroxybutyrate levels eliciting starvation-like conditions despite normal levels of food intake.

## Introduction

2,3,7,8-Tetrachlorodibenzo-*p*-dioxin (TCDD) is the prototypical member of a class of persistent environmental contaminants termed polyhalogenated aromatic hydrocarbons, which include polychlorinated dibenzodioxins (PCDDs), dibenzofurans (PCDFs) and biphenyls (PCBs)^1^. A subset of these contaminants possesses lateral chlorines that induce a diverse spectrum of aryl hydrocarbon receptor (AHR)-mediated species-, sex-, tissue-, cell- and promoter-specific responses including the dose-dependent progression of hepatic steatosis to steatohepatitis with fibrosis. Moreover, TCDD and dioxin-like PCBs are classified as an International Agency for Research on Cancer (IARC) group 1 human carcinogen while the carcinogenicity of other toxic PCDDs and PCDFs in humans is equivocal^2–4^. TCDD and related compounds are non-genotoxic with most, if not all of their effects mediated by the AHR, a ligand-activated basic helix-loop-helix PER-ARNT-SIM transcription factor that is conserved in all vertebrate species^5^. It is activated by several structurally diverse chemicals, endogenous metabolites, microbial products, and natural compounds, although the physiological ligand is unknown. Following ligand binding and the dissociation of chaperone proteins, the AHR translocates to the nucleus and dimerizes with the AHR nuclear translocator (ARNT)^6–8^. In the proposed canonical mechanism, the heterodimer binds dioxin response elements (DREs; 5’-GCGTG-3’) throughout the genome and recruits multiple coactivators to elicit differential gene expression. However, several studies also report differential gene expression independent of DREs as well as alternate AHR binding partners suggesting alternative mechanisms of gene regulation^7,9–12^.

The emergence of transcriptomics and metabolomics provides the opportunity to comprehensively assess the effects of exogenous agents on gene expression and endogenous metabolite levels. Numerous studies have examined the consequences of PCDD, PCDF, or PCB exposure on gene expression and/or metabolite levels in diverse in vivo and in vitro models^13–20^. However, few have integrated transcriptomic and metabolomic datasets with complementary histopathology to distinguish adaptive events from key responses to elucidate causative mechanisms associated with adverse outcomes^21–25^. Despite decades of research establishing the central role of the AHR in mediating the effects of TCDD and related compounds, the mechanisms leading to toxicity remain poorly understood.

Acetyl-coenzyme A (acetyl-CoA) occupies a central position in multiple metabolic pathways. As a metabolite, it straddles carbohydrate, lipid, and amino acid catabolism, and can be used as a substrate for the synthesis of fatty acids, cholesterol, and ketone bodies. Acetyl-CoA is also used as a substrate for the posttranslational modification of proteins to regulate enzyme activity, protein stability, cellular location, and to remodel chromatin via histone acetylation to control gene expression, thus linking intermediate metabolism to cellular homeostasis^26–29^. Acetyl-CoA is not membrane permeable, therefore resulting in specific cellular pools each of which support distinct activities that are independently generated within mitochondrial, peroxisomal, endoplasmic reticulum, and nucleo-cytosolic compartments. For example, the mitochondrial pool is produced by the pyruvate dehydrogenase complex (PDC), fatty acid β-oxidation, and amino acid metabolism, while the nucleo-cytosolic pool is sourced from ATP-citrate lyase (ACLY), acyl-CoA synthetase short-chain family member 2 (ACSS2), and nuclear PDC^30^. Levels of protein acetylation are directly linked to acetyl-CoA levels that fluctuate depending on intra- and extra-cellular cues that also undergo circadian regulation. This dynamic regulation of acetyl-CoA metabolism not only affects global histone modifications but also synchronizes intermediate metabolism with feeding and active/rest cycles. Consequently, acetyl-CoA is not only a metabolic intermediate but also a surrogate indicator of nutritional status that coordinates metabolic reprogramming through epigenetic regulation and posttranslational modification to sustain survival, growth and proliferation during periods of starvation, nutrient availability and metabolic stress^30,31^. For instance, nutrient starvation causing the rapid depletion of acetyl-CoA triggers autophagy due to the activation of AMPK^32^. However, the dose-dependent effects of environmental contaminants, drugs, chemicals, or natural products on intermediate metabolism, and more specifically, acetyl-CoA levels, have not been examined and warrant further investigation.

Previous studies have reported decreased ATP levels in the liver and the induction of steatosis to steatohepatitis with fibrosis following treatment with TCDD and related compounds^33–38^. Moreover, TCDD dose-dependently reprogramed glucose metabolism by switching from PKM1 to PKM2 expression resulting in reduced glycolytic flux and the redirection of accumulating upstream intermediates to other pathways to support proliferation and/or reactive oxygen species (ROS) defenses^39^. In addition, the integration of transcriptomic and metabolomic data with chromatin immunoprecipitation analyses showed TCDD dose-dependently repressed fatty acid β-oxidation^40^. In this study, we further examined the effects of TCDD on pathways associated with acetyl-CoA metabolism to test the hypothesis that acetyl-CoA levels reduced by TCDD also affected ketone body synthesis, protein acetylation and β-hydroxybutyrylation, and AMPK activation. Our analysis found TCDD lowered hepatic acetyl-CoA and β-hydroxybutyrate levels. Accordingly, total hepatic protein acetylation and hydroxybutyrylation levels were reduced with increased levels of activated AMPK, suggesting the induction of a starvation-like phenotype in the liver despite unaffected levels of food intake. These results indicate that in addition to differential gene expression mediated by the AHR, TCDD can also elicit secondary effects by disrupting acetyl-CoA homeostasis.

## Results

### Gross morphology and histopathology

In agreement with previously reported findings, 30 μg/kg TCDD decreased terminal body weight by ~ 14% (Supplementary Fig. 1A), despite no significant change in daily food intake^23^. Absolute liver weights increased 12–30% between 0.3 and 30 μg/kg TCDD (Supplementary Fig. 1B), while relative liver weight dose-dependently increased 7–44% between 0.3 and 30 μg/kg TCDD (Supplementary Fig. 1C). Absolute and relative gonadal white adipose tissue weights have previously been reported to decrease 46% and 28%, respectively, at 30 μg/kg TCDD with no change in terminal brown adipose tissue weights^41^. Although a modest increase in serum ALT levels was observed following oral gavage with 30 µg/kg TCDD every 4 days for 28 days for a total of 7 treatments (Supplementary Fig. 1D), previous studies eliciting comparable effects exhibited no evidence of overt toxicity or body weight loss > 15%^41,42^.

Hepatic steatosis, immune cell infiltration, fibrosis, and bile duct proliferation have previously been reported to be dose-dependently induced following oral gavage with TCDD every 4 days for 28 days^33^. Specifically, there was evidence of hepatocyte vacuolization (fatty change) with minimal to slight hepatocyte necrosis at 3 μg/kg TCDD and immune cell infiltration after doses of ≥ 3 μg/kg TCDD after 28 days of exposure, with F4/80 staining confirming the presence of macrophages. At 30 μg/kg, bile duct proliferation was observed along with picro-Sirius red staining for collagen and inflammation surrounding the bile ducts (pericholangitis). Collectively, the gross morphology, histopathology, and ALT results suggest the effects on gene expression, protein levels and metabolite levels do not induce overt toxicity following oral gavage with TCDD every 4 days for 28 days.

### MS analysis of acyl-CoA and CoA levels

**Figure 1:**
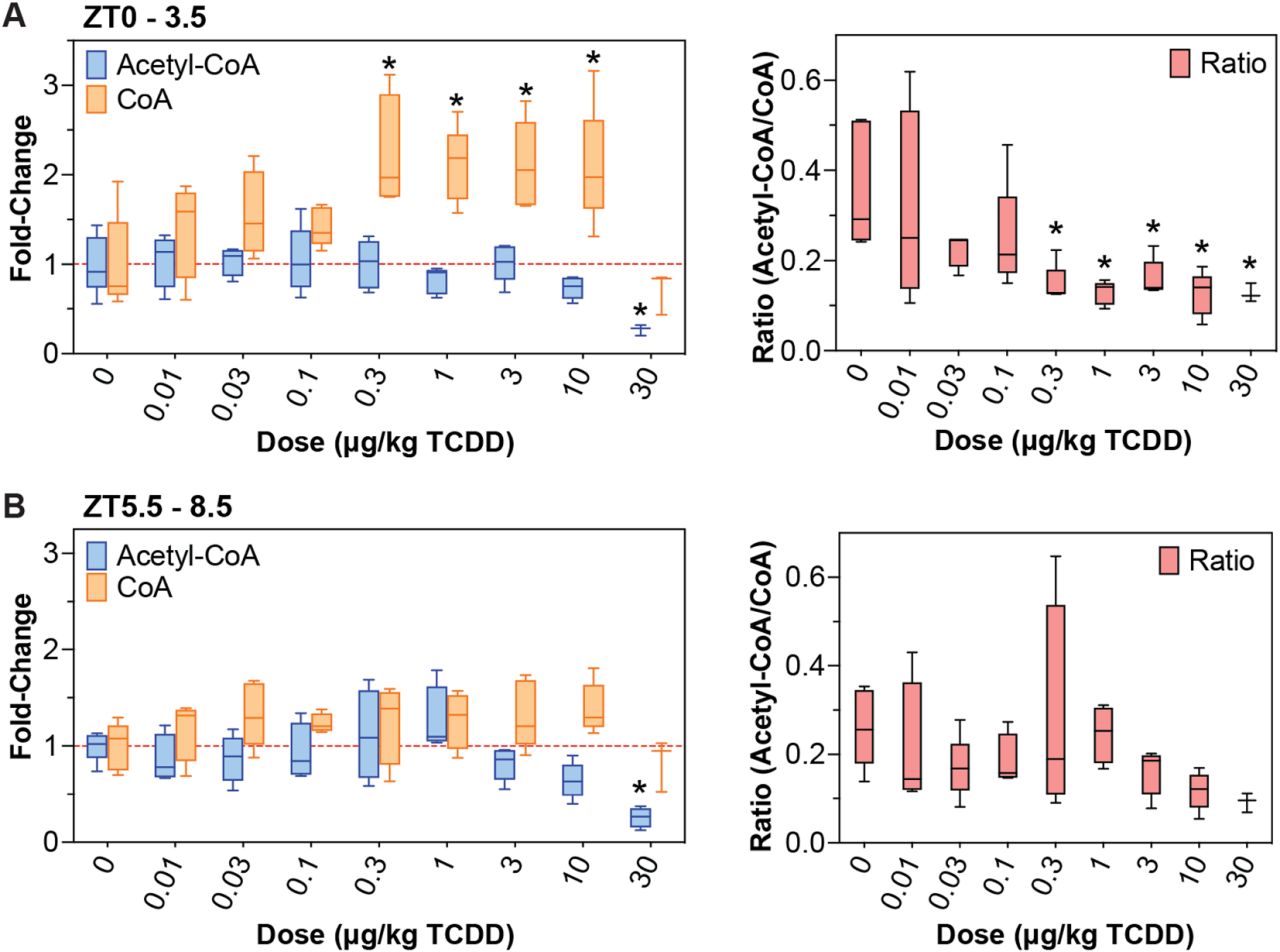
Hepatic acetyl-CoA and coenzyme A (CoA) levels assessed in mice using targeted liquid chromatography tandem mass spectrometry. Mice were gavaged with TCDD (or sesame oil vehicle) between zeitgeber time (ZT) 0–1. Samples were collected at (**A**) ZT 0–3.5 h, or (**B**) ZT 5.5–8.5 h. Asterisk (*) denotes a significant change (p ≤ 0.05) as determined using a one-way ANOVA and Dunnett’s *post-hoc* analysis.

Previously reported untargeted metabolomics identified a dose-dependent decrease in hepatic acetyl-CoA levels^40^. These results were confirmed by targeted analysis with internal standards (Fig. 1A,B). Due to circadian regulation of carbohydrate, lipid, and protein metabolism, targeted analysis assessed samples collected in the morning (zeitgeber time [ZT] 0–3.5) and afternoon (ZT5.5–8.5) samples. Acetyl-CoA levels in controls (74.3 ± 6.4 nmol/g wet tissue; or 2.4 ± 0.21 nmol/mg total protein) did not change between morning and afternoon cohorts, and were comparable to previously reported levels^43,44^. TCDD lowered morning and afternoon acetyl-CoA levels 3.8- and 3.4-fold, respectively. In contrast, CoA levels dose-dependently increased 2.2-fold at lower TCDD doses but decreased 2.9-fold at 30 μg/kg TCDD in morning samples. Although TCDD did not affect afternoon CoA levels, the acetyl-CoA/CoA ratio exhibited a decreasing trend due to lowering acetyl-CoA levels (Fig. 1B).

### Glycolysis and PDH as a source of Acetyl-CoA

**Figure 2:**
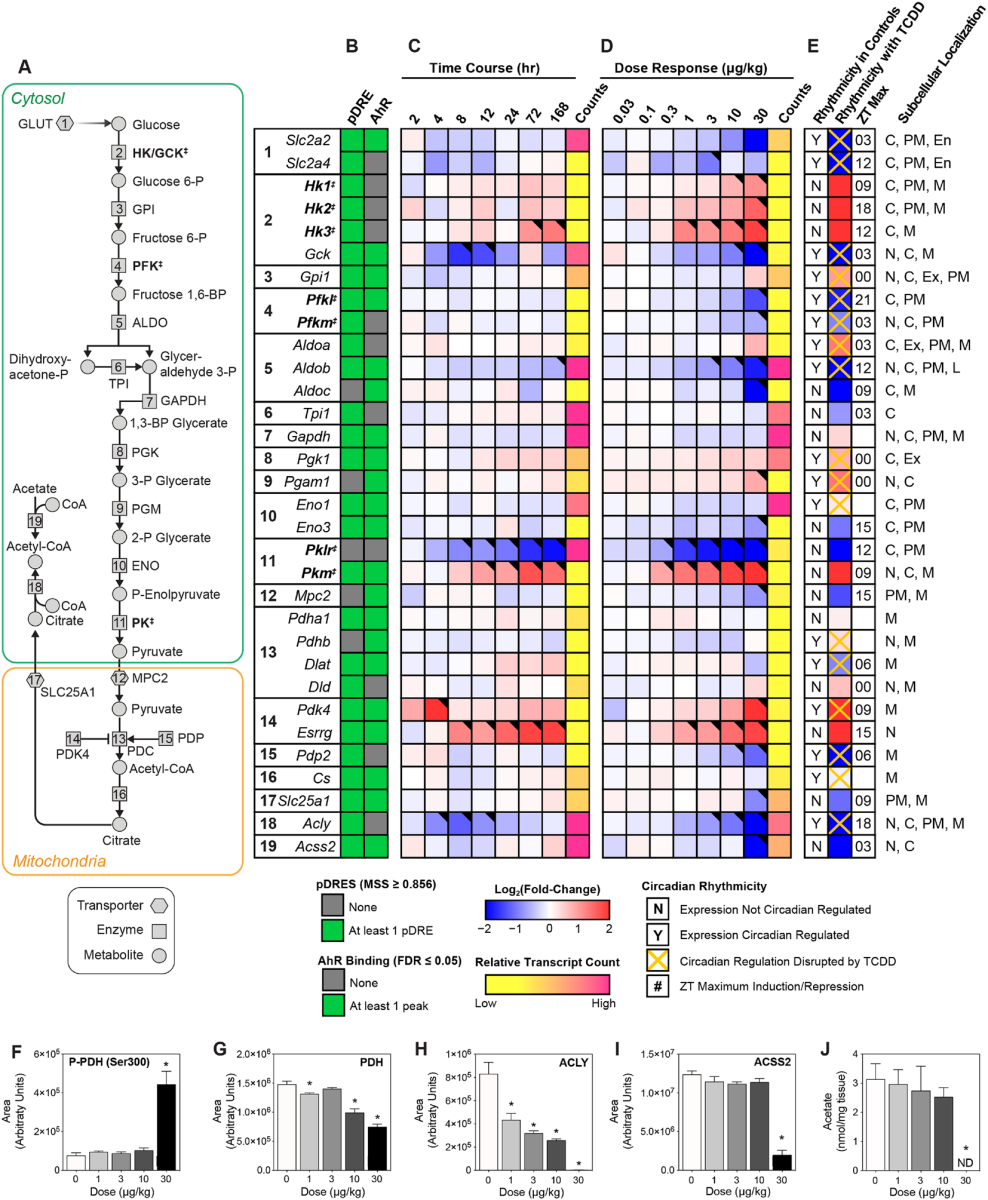
Glucose metabolism as a source of acetyl-CoA. Differential gene expression was assessed using RNA-seq. (**A**) The glycolysis pathway with regulated steps denoted with a double dagger (‡). (**B**) Computational identification of putative dioxin response elements (pDREs), and the detection of hepatic AhR genomic binding in ChIPseq analysis 2 h after oral gavage of 30 μg/kg TCDD. Genes are listed by the official symbol as designated in the mouse genome informatics (MGI) database. (**C**) Time-dependent hepatic expression of glycolysis-related genes (n = 3) following a single bolus gavage of 30 μg/kg TCDD. (**D**) Hepatic dose-dependent gene expression (n = 5) following oral gavage with TCDD every 4 days for 28 days. (**E**) Diurnal regulated gene expression denoted with a “Y”. An orange ‘X’ indicates oscillating gene expression was abolished following oral gavage with 30 μg/kg TCDD every 4 days for 28 days. ZT indicates the time of maximum induction/repression (P1(t) > 0.8). Counts represents the maximum number of raw reads for any treatment group. Low transcript counts (< 500 reads) are denoted in yellow with high transcript counts (> 10,000) denoted in pink. Differential gene expression with a posterior probability (P1(t)) > 0.80 are depicted with a black triangle in the upper right corner of the tile. Capillary electrophoresis was used to assess (**F**) phosphorylated pyruvate dehydrogenase (Ser300), (**G**) total pyruvate dehydrogenase, (**H**) ATP-citrate lyase, and (**I**) acyl-CoA synthetase short chain family member 2 protein levels in total lysate prepared from liver samples harvested between ZT0-3 (n = 3). (**J**) Hepatic levels of acetate, a precursor for acetyl-CoA, were assessed. Bar graphs denote the mean ± SEM. Significance (*p ≤ 0.05) was determined using a one-way ANOVA followed by Dunnett’s *post-hoc* analysis. The heatmap was created using R (v4.0.4). Plots were created using GraphPad Prism (v8.4.3). The biochemical reaction was created using Adobe Illustrator (v25.2).

We next examined changes associated with carbohydrate catabolism, a primary source of acetyl-CoA (Fig. 2A). Figure 2 summarizes the TCDD-induced changes in the expression of glycolytic genes following oral gavage every 4 days for 28 days. The presence of a computationally identified putative DRE (pDRE) and AHR genomic binding (ChIPseq) data, as well as time course, diurnal regulated dose–response, and diurnal regulated RNAseq gene expression data are included in all heatmaps (Fig. 2B–E). Gene expression changes reported in the text represent the maximum fold-change determined in the diurnal regulated gene expression dataset with the corresponding ZT.

Glucokinase (*Gck*), aldolase B (*Aldob*), liver/red blood cell pyruvate kinase (*Pklr*), and pyruvate dehydrogenase phosphatase catalytic subunit 2 (*Pdp2*) showed time and dose-dependent repression, while hexokinase 1, 2, and 3 (*Hk1*, *2,* and *3*), muscle pyruvate kinase (*Pkm*), and pyruvate dehydrogenase kinase 4 (*Pdk4*) were induced. *Hk2* has recently been reported to be positively regulated by the AHR in human U2OS and 143B osteosarcoma cells, as well as human HCT116 colon cancer cell lines^45^. Glucose transporters GLUT2 (*Slc2a2*) and GLUT9 (*Slc2a9*), which are responsible for transporting glucose primarily in the liver^46^, were also dose-dependently repressed. Only *Slc2a2, Slc2a9, Gck*, *Aldob*, *Pkm* and *Pdk4* exhibited AHR genomic enrichment 2 h after treatment with 30 μg/kg TCDD. TCDD and related polychlorinated biphenyls (PCB126 and 118) have been reported to specifically induce nuclear and cytosolic pyruvate kinase muscle isoform 2 (*Pkm2*)^39,47,48^. Nuclear PKM2 has been implicated in gene regulation while the cytosolic dimer is associated with the Warburg effect with lower catalytic activity compared to PKM1 and PKLR^49,50^. Following TCDD-induced PKM isoform switching, accumulating upstream glycolytic intermediates are redirected to the pentose phosphate pathway and serine/folate biosynthesis for biomass and NADPH production to support proliferation and the biosynthesis and recycling of glutathione, depending on the microenvironment^51^. Consequently, the reduced glycolytic flux following PKM2 induction and PKLR repression is consistent with decreased acetyl-CoA levels.

Acetyl-CoA levels are also affected by the activity of the pyruvate dehydrogenase complex (PDC). PDC is posttranslationally regulated by phosphorylation, acetylation, and succinylation^52^. The 131.1-fold induction of *Pdk4* and the 4.8-fold repression of pyruvate dehydrogenase phosphatase 2 (*Pdp2*) suggest phosphorylation of the E1α subunit of pyruvate dehydrogenase (PDH) at Ser300. PDK4 is reported to primarily phosphorylate Ser293 and Ser300 sites of the PDH^53^, and was confirmed by capillary electrophoresis with the increase in phosphorylated PDH at 30 μg/kg TCDD that could not be attributed to overall higher total hepatic PDH levels (Fig. 2F,G). Moreover, estrogen-related receptor gamma (*Esrrg*), which induces PDK4 activity under hypoxic conditions^54^, was induced 3.9-fold. ACLY and ACSS2, which produce cytosolic pools of acetyl-CoA from citrate and acetate, were also repressed 4.3- and 8.8-fold, repression, respectively (Fig. 2H,I). Total hepatic acetate levels also decrease, but only at 30 μg/kg TCDD (Fig. 2J).

### Coenzyme A biosynthesis

**Figure 3:**
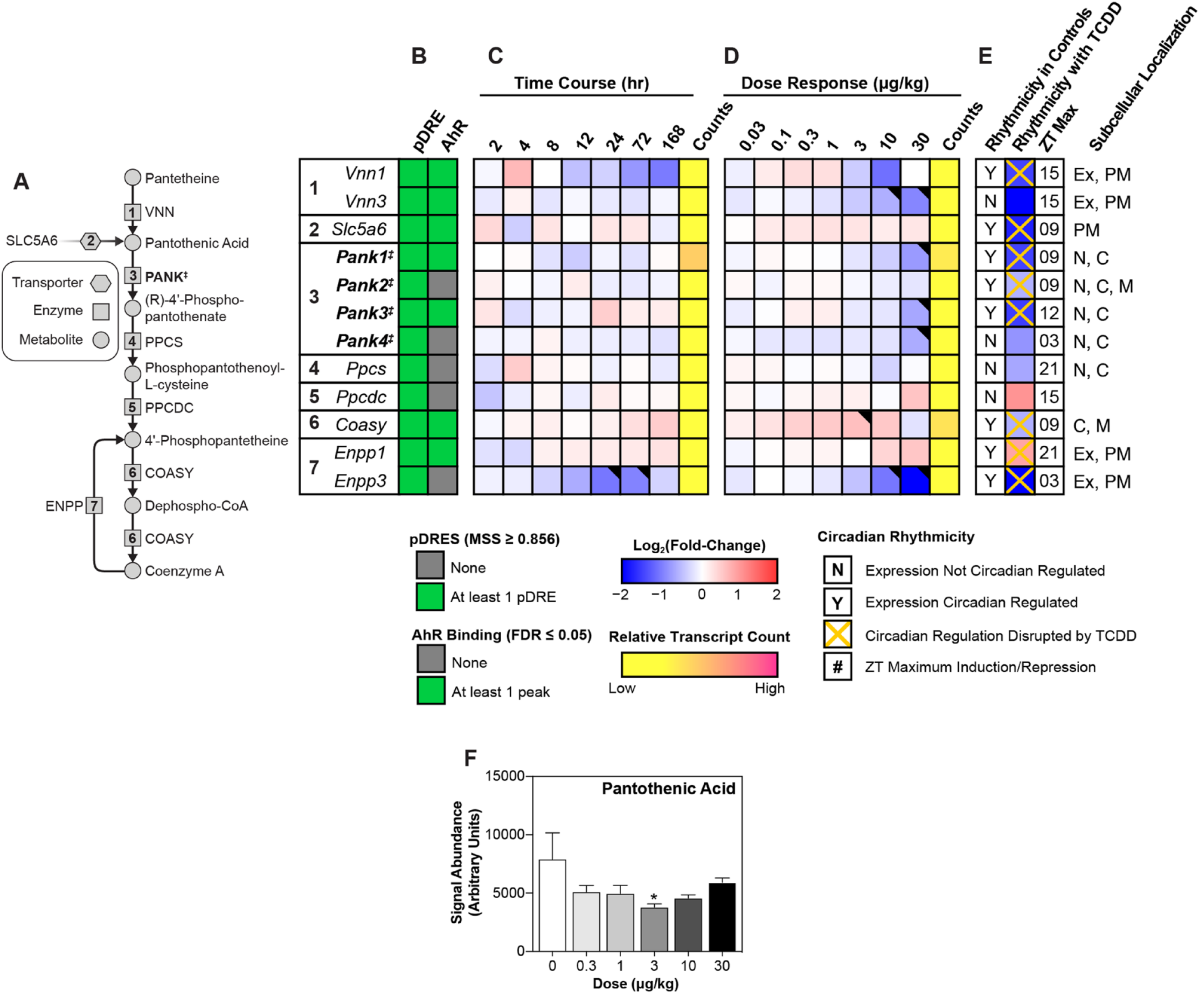
Biosynthesis of coenzyme A (CoA). Differential gene expression pertaining to CoA biosynthesis was assessed using RNA-seq. (**A**) The CoA biosynthesis pathway is shown with regulated steps denoted with a double dagger (‡). (**B**) Computational identification of putative dioxin response elements (pDREs), and the detection of hepatic AhR genomic binding in ChIPseq analysis 2 h after oral gavage of 30 μg/kg TCDD. Genes are listed by the official symbol as designated in the mouse genome informatics (MGI) database. (**C**) Time-dependent hepatic expression of glycolysis-related genes (n = 3) following a single bolus gavage of 30 μg/kg TCDD. (**D**) Hepatic dose-dependent gene expression (n = 5) following oral gavage with TCDD every 4 days for 28 days. (**E**) Diurnal regulated gene expression denoted with a “Y”. An orange ‘X’ indicates oscillating gene expression was abolished following oral gavage with 30 μg/kg TCDD every 4 days for 28 days. ZT indicates the time of maximum induction/repression (P1(t) > 0.8). Counts represent the maximum number of raw reads for any treatment group. Low transcript counts (< 500 reads) are denoted in yellow with high transcript counts (> 10,000) denoted in pink. Differential gene expression with a posterior probability (P1(t)) > 0.80 are depicted with a black triangle in the upper right corner of the tile. (**F**) Hepatic levels of pantothenic acid were assessed using target LC–MS/MS. Bar graphs denote the mean ± SEM. Significance (*p ≤ 0.05) was determined using a one-way ANOVA followed by Dunnett’s post-hoc analysis. The heatmap was created using R (v4.0.4). Plots were created using GraphPad Prism (v8.4.3). The biochemical reaction was created using Adobe Illustrator (v25.2).

Acetyl-CoA biosynthesis is dependent on the availability of coenzyme A (CoA), an ubiquitous cofactor synthesized from the essential vitamin pantothenate^55^ (Fig. 3A). CoA is crucial to many metabolic pathways including the tricarboxylic acid and β-oxidation cycles. Biosynthesis begins with intestinal absorption of either dietary or microbial-derived pantothenate absorbed from the gut, after which it is transported to peripheral tissues and imported into cells via the sodium-dependent multivitamin transporter (*Slc5a6*; repressed 3.3-fold), or by passive diffusion^55,56^ (Fig. 3B–E). Pantothenate kinases (*Pank1-4*; repressed 2.8-, 1.5-, 2.9-, and 1.8-fold, respectively) phosphorylate pantothenic acid to yield 4′-phosphopantothenate which is then converted to 4′-phosphopantothenoylcysteine by 4′-phosphopantothenoylcysteine synthetase (*Ppcs*; repressed 1.6-fold), followed by decarboxylation mediated by 4′-phosphopantothenoylcysteine decarboxylase (*Ppcdc*; induced 2.1-fold) to yield 4′-phosphopantetheine. Bifunctional coenzyme A synthase (*Coasy*; repressed 1.5-fold) catalyzes the last two reactions to first yield dephospho-coenzyme A, and finally CoA. TCDD elicited a non-monotonic decrease in hepatic pantothenic acid levels that was only significant at 3 μg/kg TCDD (Fig. 3F). This increase in CoA (Fig. 1) cannot be attributed to CoA biosynthesis gene induction or increased hepatic levels of pantothenic acid (Fig. 3).

### Ketone body formation

**Figure 4:**
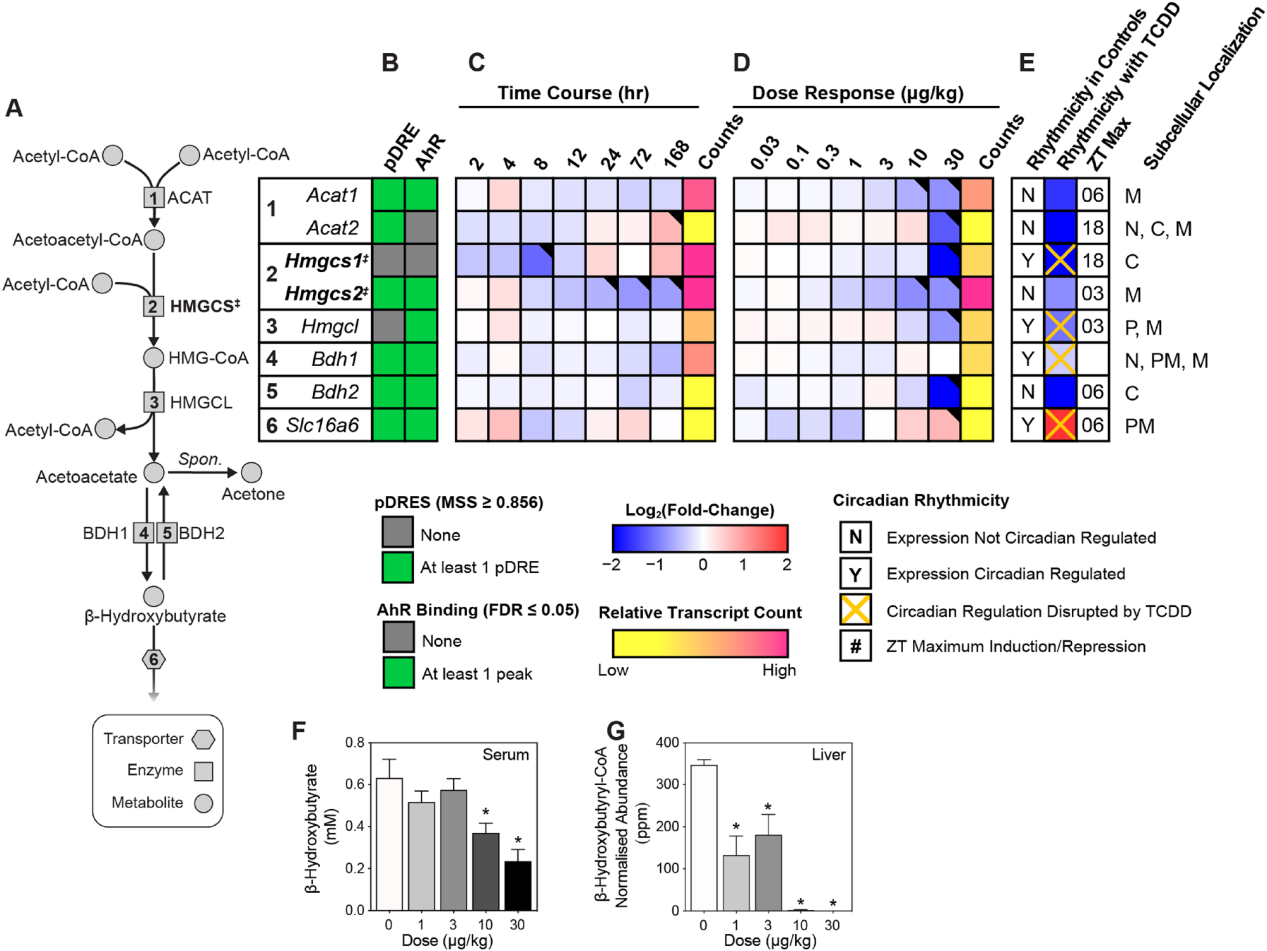
Effects of TCDD on ketone body gene expression and levels. Differential gene expression pertaining to ketone body biosynthesis was assessed using RNA-seq. (**A**) The ketone body biosynthesis pathway with regulated steps denoted with a double dagger (‡). (**B**) Computational identification of putative dioxin response elements (pDREs), and the detection of hepatic AhR genomic binding in ChIPseq analysis 2 h after oral gavage of 30 μg/kg TCDD. Genes are listed by the official symbol as designated in the mouse genome informatics (MGI) database. (**C**) Time-dependent hepatic expression of glycolysis-related genes (n = 3) following a single bolus gavage of 30 μg/kg TCDD. (**D**) Hepatic dose-dependent gene expression (n = 5) following oral gavage with TCDD every 4 days for 28 days. (**E**) Diurnal regulated gene expression denoted with a “Y”. An orange ‘X’ indicates oscillating gene expression was abolished following oral gavage with 30 μg/kg TCDD every 4 days for 28 days. ZT indicates the time of maximum induction/repression (P1(t) > 0.8). Counts represent the maximum number of raw reads for any treatment group. Low transcript counts (< 500 reads) are denoted in yellow with high transcript counts (> 10,000) denoted in pink. Differential gene expression with a posterior probability (P1(t)) > 0.80 are depicted with a black triangle in the upper right corner of the tile. (**F**) Serum β-hydroxybutyrate and (**G**) hepatic β-hydroxybutyryl-CoA levels were assessed using a commercially available kit and targeted LC–MS/MS, respectively. Bar graphs denote the mean ± SEM. Significance (*p ≤ 0.05) was determined using a one-way ANOVA followed by Dunnett’s *post-hoc* analysis. The heatmap was created using R (v4.0.4). The biochemical reaction was created using Adobe Illustrator (v25.2).

In the fed state, cells can utilize acetyl-CoA for fatty acid biosynthesis, protein acetylation, cholesterol biosynthesis, and energy production via the TCA cycle^30^. Under fasting conditions, however, metabolic reprogramming shifts acetyl-CoA into mitochondrial oxidative catabolism to support the synthesis of either ATP or the easily transported ketone bodies, acetoacetate, β-hydroxybutyrate, and acetone^30,57^. Biosynthesis consumes two acetyl-CoAs for each ketone body (Fig. 4A), beginning with the formation of acetoacetyl-CoA via acyl-CoA:cholesterol acyltransferase (*Acat1* and *2*, repressed 3.1- and 4.3-fold, respectively)^58,59^. An additional acetyl-CoA undergoes condensation with acetoacetyl-CoA to form hydroxymethylglutaryl-CoA catalyzed by the rate-limiting hydroxymethylglutaryl (HMG)-CoA synthase (HMGCS). *Hmgcs1* and *2* were repressed 10.4- and 1.9-fold, respectively, by TCDD (Fig. 4B–E). The HMG-CoA intermediate can then be metabolized into ketone bodies or shunted to the mevalonate pathway for cholesterol metabolism. However, TCDD repressed gene expression associated with de novo cholesterol biosynthesis^14,60^. Although HMG-CoA can be lysed to acetoacetate, HMG-CoA lyase (*Hmgcl*) was repressed 2.1-fold. In addition, TCDD repressed β-hydroxybutyrate dehydrogenase 1 (*Bdh1*) 3.2-fold thus limiting the oxidation of acetoacetate to β-hydroxybutyrate. β-Hydroxybutyrate dehydrogenase 2 (*Bdh2*) which catalyzes the reverse reaction and the formation of acetoacetate, was also repressed 106.4-fold. Although ketone bodies can be exported^61^, the induction of *Slc16a6*, the ketone body transporter, was negligible. Furthermore, following 30 μg/kg TCDD, serum β-hydroxybutyrate were reduced 2.5-fold, while hepatic β-hydroxybutyryl-CoA levels were undetectable (Fig. 4F,G). Overall, gene expression involved in ketone body biosynthesis was repressed, and for the most part, exhibited AHR genomic binding in the presence of a pDRE. TCDD also elicited a dramatic dose-dependent decrease in β-hydroxybutyrate levels in serum and hepatic extracts suggesting that decreased hepatic acetyl-CoA levels were not due to cholesterol nor ketone body biosynthesis.

### Protein acetyl and β-Hydroxybutyryl posttranslational modifications

**Figure 5:**
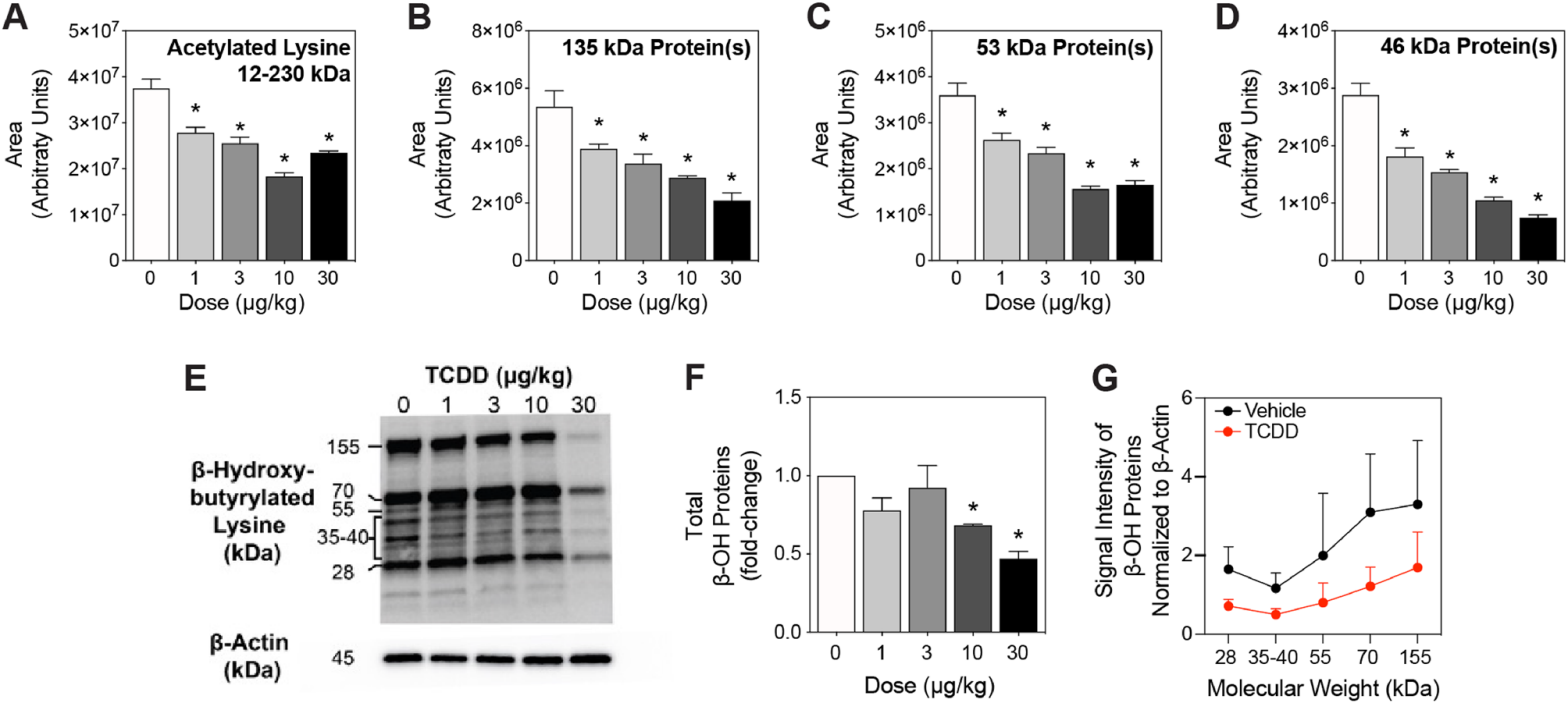
(**A**) Total lysine-specific acetylated proteins were assessed in livers of mice (n = 3–5) by capillary electrophoresis. Mice were orally gavaged every 4 days for 28 days with 0.01, 0.03, 0.1, 0.3, 1, 3, 10 or 30 μg/kg TCDD prior to tissue collection. (**B–D**) Area under the three most prominent peaks for total proteins with acetylated lysine was quantified. (**E,F**) Total β-hydroxybutyrylated proteins were assessed by traditional Western blot (n = 3). Depicted is a representative Western blot of total β-hydroxybutyrylated proteins, as well as β-actin from total protein extracts. The full Western blots can be found in Supplementary Fig. 2. (**G**) The signal intensity of total β-hydroxybutyrylated proteins at various molecular weights for vehicle and 30 μg/kg treatment groups quantified using ImageJ as outlined in materials and methods. Bar graphs denote the mean ± SEM. Significance (*p ≤ 0.05) was determined using a one-way ANOVA followed by Dunnett’s *post-hoc* analysis. Plots were created using GraphPad Prism (v8.4.3).

In addition to being metabolic intermediates, acetyl-CoA and β-hydroxybutyrate are also used as substrates for posttranslational modifications (PTMs). Approximately 90% of eukaryotic proteins undergo different types of reversible PTM, with moieties typically added onto lysine residues to regulate enzymatic activity, alter protein stability, and change protein localization and interactions with other proteins^62,63^. The level for protein acetylation and β-hydroxybutyrylation are dependent on the levels acetyl-CoA and β-hydroxybutyryl-CoA, respectively, that serve as source of the donor group^64^. β-Hydroxybutyrate is first activated to β-hydroxybutyryl-CoA by acyl-CoA short-chain synthetases such as ACSS2^63^, which was repressed 9.1-fold in the present study. Consequently, capillary electrophoresis and Western blotting were used to investigate the effect of TCDD on the levels of total hepatic lysine-specific protein acetylation and β-hydroxybutyrylation (Fig. 5). TCDD dose-dependently decreased the level of total acetylated (Fig. 5A) and β-hydroxybutyrylated proteins in hepatic extracts prepared from treated mice. 135, 53, and 46 kDa protein(s) were dose-dependently decreased in acetylation (Fig. 5B–D), while β-hydroxybutyrylation was decreased in 28 to 155 kDa proteins (Fig. 5E–G). Reductions in the levels of acetylated and β-hydroxybutyrylated hepatic proteins are consistent with lower levels of acetyl-CoA and β-hydroxybutyrate.

### AMPK activation

**Figure 6:**
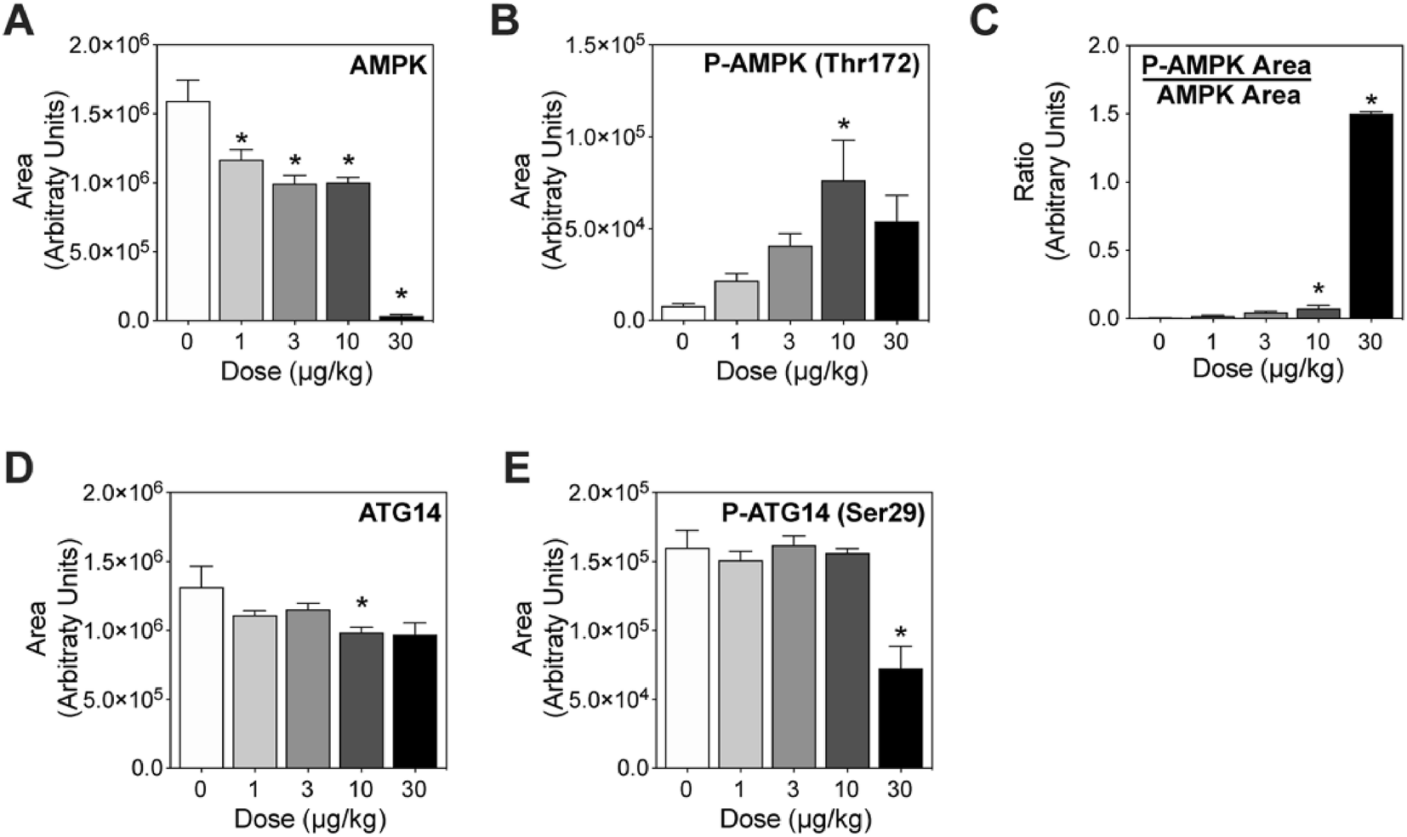
Markers of AMPK activation. Mice (n = 3–5) were orally gavaged every 4 days for 28 days with TCDD prior to tissue collection. Protein levels were assessed in hepatic extracts using capillary immunoassay analysis. (**A**) Total AMPK and (**B**) phosphorylated AMPK (P-AMPK) and (**C**) the P-AMPK/AMP ratio two were assessed. Capillary immunoassay analysis of (**D**) total ATG14 and (**E**) phosphorylated ATG14 at serine 29 (P-ATG14 Ser29), a marker of P-AMPK activation. Bar graphs denote the mean ± SEM. Significance (*p ≤ 0.05) was determined using a one-way ANOVA followed by Dunnett’s *post-hoc* analysis. Plots were created using GraphPad Prism (v8.4.3).

Cellular levels of acetyl-CoA provide a surrogate measure of the current metabolic state^65^. AMP-activated protein kinase (AMPK) senses energy status by monitoring changes in the AMP:ATP and ADP:ATP ratios^66^. Phosphorylation of the catalytic subunit of the trimeric AMPK protein is required for activation^66^. In the present study, TCDD dose-dependently decreased total hepatic AMPK, with a precipitous decrease at 30 μg/kg TCDD that corresponded with an increase in phosphorylated AMPK (P-AMPK) and the phosphorylated AMPK:total AMPK ratio (Fig. 6A–C). In response to low energy status, P-AMPK activates catabolic pathways to increase energy production. For example, in response to low energy levels, P-AMPK induces autophagy in an attempt to compensate for the lack of nutrients and provide substrates for catabolism. Autophagy can be independently regulated by P-AMPK following phosphorylation of the ULK complex that can then bind and phosphorylate autophagy related 14 (ATG14), a marker of cellular autophagy activity. Total and phosphorylated ATG14 levels (Fig. 6D,E) in hepatic extracts show that both ATG14 and phosphorylated ATG14 decreased following treatment with 30 μg/kg TCDD. Further investigation of P-AMPK activity on other known targets including acetyl-CoA carboxylase and RAPTOR were also equivocal suggesting P-AMPK was not activated despite phosphorylation.

## Discussion

This is one of the first studies to examine acetyl-CoA levels in a model of TCDD-induced steatohepatitis with fibrosis. Acetyl-CoA is a central metabolite associated with several catabolic pathways including glycolysis, the tricarboxylic acid cycle, β-oxidation, and amino acid metabolism (e.g. lysine, valine, leucine and isoleucine), as well as a substrate used for the synthesis of fatty acids, cholesterol, and ketone bodies^30,67^. Previous studies have shown that TCDD dose-dependently caused metabolic reprograming affecting both glycolysis and fatty acid β-oxidation^22,40^. The present study further investigated TCDD-elicited metabolic reprogramming associated with acetyl-CoA metabolism. Our results indicate TCDD dose-dependently decreased the ratio of acetyl-CoA/CoA, an overall indicator of cellular energy status, and therefore additional enzymatic reactions and pathways associated with acetyl-CoA homeostasis were examined.

TCDD elicits a dose-dependent induction of pyruvate kinase isoform 2 (PKM2) which is reported to decrease glycolytic flux and shunt accumulating glycolytic intermediates to other pathways resulting in lower glucose-6-phosphate and fructose-6-phosphate levels^22,39^. Pyruvate, the end product of glycolysis, can then be oxidized to acetyl-CoA by the PDC composed of the (i) pyruvate dehydrogenase (E1), (ii) acetyl transferase (E2), and (iii) dihydrolipoyl dehydrogenase (E3) subunits. PDC is regulated by phosphorylation and dephosphorylation of the E1 subunit by pyruvate dehydrogenase kinases (PDKs) and pyruvate dehydrogenase phosphatases (PDPs), respectively. Specifically, E1 phosphorylation at Ser232, Ser293, and Ser300, inhibits PDC activity and reduces acetyl-CoA production^68^. TCDD dose-dependently induced *Pdk4* and repressed *Pdp2*, while estrogen-related receptor γ (*ERRγ*) was induced which is associated with the induction of PDK4 in hepatoma cell lines under hypoxic conditions^54^. In addition, TCDD repressed expression of the highly expressed *Acss2* and *Acly*, two important enzymes that contribute to the cytosolic acetyl-CoA pool. In previous studies, TCDD treatment increased hepatic levels of the TCA cycle intermediate oxaloacetate^39^. Increased oxalacetate levels may be due to depleted acetyl-CoA levels since both are required for the synthesis of the TCA cycle intermediate citrate. Collectively, metabolic reprogramming due to PKM2 induction, the inhibition of PDC following E1 phosphorylation, and the repression of *Acss2* and *Acly*, two sources of acetyl-CoA produced from free acetate and citrate, respectively, are consistent with the overall reduction in acetyl-CoA. Missing in this analysis is an examination of the effects of TCDD on amino acid metabolism, another minor source of acetyl-CoA that is currently being investigated in a companion study.

Lower acetyl-CoA levels are indicators of insulin resistance, obesity, and cancer^69,70^. For example, squamous cell carcinomas and adenocarcinomas express high levels of PDK1 with evidence of PDC inactivation during metastasis^71,72^. Coincidentally, the International Agency for Research on Cancer (IARC) classifies TCDD as a human carcinogen^3^. Long-term treatment of rodents with TCDD leads to the development of tumors in multiple tissues including the liver, although the carcinogenic mechanism has not been resolved^73^. In humans, TCDD exposure has been associated with obesity, insulin resistance, and diabetes^74–76^. The doses used here take into consideration the relatively short duration of this study compared to the lifelong exposure of humans to diverse AhR ligands, the bioaccumulative nature of halogenated AhR ligands, and the differences in the half-life of TCDD between humans (1–11 years^77,78^) and mice (8–12 days^79^). The same dose levels and treatment regimen have also been used in previous studies and recently shown to approach steady-state levels^14,22,41,80–82^. Specifically, orally gavaging mice with 0.01 to 30 μg/kg TCDD every 4 days for 28 days resulted in mouse hepatic tissue levels that span human background serum concentrations reported in the United States, Germany, Spain, and the United Kingdom as well as serum levels in Viktor Yushchenko 4–39 months following intentional poisoning^22^. Collectively, this suggests that metabolic reprogramming resulting in reduced acetyl-CoA levels following persistent AhR activation by TCDD may have a role in the etiology of cancer and metabolic diseases such as insulin resistance and diabetes.

Few studies have assessed how liver disease progression affects acetyl-CoA and acetyl-CoA-derived intermediates. Studies examining fatty liver in mice on high-fat diets (HFD) have reported reduced pyruvate dehydrogenase complex activity, as well as *Pdk2* and *Pdk4* induction^83^. Lower β-hydroxybutyric acid in the serum for NAFLD patients also suggests fatty liver may impair ketogenesis^84^. Studies also report conflicting results regarding hepatic acetyl-CoA levels in HFD-induced NAFLD but this can likely be attributed to significant differences in models, HFDs and study duration^85,86^. Nevertheless, the possibility that TCDD does not act directly, and that hepatic fat accumulation reduces acetyl-CoA levels cannot be excluded given the results.

Under conditions of lower acetyl-CoA levels, intracellular ATP levels would also decrease and trigger the activation of AMPK, a ubiquitous sensor of cellular energy and nutrient status. AMPK monitors the AMP/ATP ratio, and is activated following phosphorylation to restore energy homeostasis by turning on catabolic pathways that provide substrates for ATP production while switching off biosynthetic pathways and other nonessential processes that consume energy^87^. Accordingly, TCDD dose-dependently increased the phosphorylated AMPK (P-AMPK, active form)/unphosphorylated AMPK (inactive form) ratio. However, known targets of P-AMPK such as acetyl-CoA carboxylase, a regulated enzyme in fatty acid biosynthesis, and downstream targets such as ATG14, a marker of autophagy, did not exhibit phosphorylation but were instead transcriptionally repressed by TCDD. Likewise, there was no evidence of RAPTOR phosphorylation that would induce the dissociation of mTOR from lysosomes to reduce biomass production in support of cell growth and proliferation. ATG14, a key regulator of autophagy, was also not phosphorylated and consequently, macromolecules were not available for catabolism to generate ATP. Overall, the lower acetyl-CoA/CoA ratio following TCDD treatment is consistent with the inability of P-AMPK to restore energy homeostasis. Further studies are needed to investigate why P-AMPK did not activate autophagy under conditions of low acetyl-CoA levels.

In addition to being a substrate for fatty acid and cholesterol synthesis which are repressed by TCDD^14,88^, acetyl-CoA can be used as a substrate for hepatic ketone body production during starvation or low circulating glucose levels^30^. The present study showed that after 6 h of fasting, hepatic and serum levels of β-hydroxybutyrate were dose-dependently decreased by TCDD, consistent with lower levels of acetyl-CoA production. Furthermore, acetyl-CoA and β-hydroxybutyrate-CoA can be used as substrates for the reversible posttranslational modification of proteins that can affect protein structure, enzymatic activity, cellular location, and protein–protein interactions^89,90^. Protein acetylation and β-hydroxybutyrylation PTMs are particularly important in gluconeogenesis, glycolysis, the TCA and urea cycles, glycogen metabolism, and fatty acid metabolism. Consequently, these intermediates can exert a signaling function that links metabolism and metabolite levels to gene expression^63,90,91^. In the present study, TCDD dose-dependently decreased total hepatic protein acetylation and β-hydroxybutyrylation PTMs which has been shown to correlate with acetyl-CoA and β-hydroxybutyrate levels^92^. This extends the potential effects of TCDD beyond direct AHR-mediated effects on gene expression to later indirect consequences due the disruption of protein PTM.

In summary, TCDD elicited dose-dependent hepatic metabolic reprogramming by direct AHR-mediated action on gene expression, while also causing later indirect effects by altering protein acetylation and β-hydroxybutyrylation. Specifically, the present study presents further evidence of TCDD-mediated metabolic reprograming events that contribute to an energy crisis as demonstrated by the reduced levels of intracellular acetyl-CoA and the induction of P-AMPK. We have previously shown that exposure to TCDD in mice results in the metabolic inhibition of glycolysis and fatty acid β-oxidation, pathways that replenish acetyl-CoA levels when cell energy stores are low. The present study provides evidence that TCDD impeded glycolysis not only due to PKM isoform switching, but also through the inactivation of the PDC, the gateway between glycolysis and the TCA cycle. Further investigation is necessary to elucidate the paradoxical energy dysregulation induced by TCDD.

## Materials and methods

### Animal treatment

Mice were housed and treated as previously described^40^. Briefly, postnatal day (PND) 25 male C57BL/6 mice, obtained from Charles River Laboratories (Kingston, NY), were house in Innovive Innocages (San Diego, CA) containing ALPHA-dri bedding (Shepherd Specialty Papers, Chicago, IL). Cages were housed in a 12 h/12 h light/dark cycle and at 23 °C environment with 30–40% humidity. Harlan Teklad 22/5 Rodent Diet 8940 (Madison, WI) and Aquavive water (Innovive) were provided ad libitum. A TCDD stock was prepared as previously described^40^. On PND28, mice were orally gavaged at the start of the light cycle (zeitgeber [ZT] 0) with 0.1 ml sesame oil vehicle (Sigma-Aldrich, St. Louis, MO) or 0.03, 0.1, 0.3, 1, 3, 10, and 30 μg/kg body weight TCDD every 4 days for 28 days for a total of 7 treatments. This dosing regimen was selected to approach steady state levels given the 8–12 day half-life of TCDD in mice^93^. Comparable treatment has been used in previous studies^14,22,23,25,40,81,94^. Following 28 days, mice were weighed and euthanized. Serum and liver tissues were collected and immediately flash-frozen in liquid nitrogen and stored at -80 °C. This study was conducted in accordance with relevant guidelines and regulations. All animal procedures were approved by the Michigan State University (MSU) Institutional Animal Care and Use Committee (IACUC; PROTO201800043) and meet the ARRIVE guidelines.

### Liquid chromatography tandem mass spectrometry

Previously published untargeted liquid chromatography tandem mass spectrometry data was used to assess hepatic β-hydroxybutyryl-CoA levels^40^. Dataset was accessed through the NIH Metabolomics Workbench (ST001379). Targeted acetyl-CoA and coenzyme A samples were measured on a Xevo G2-XS QTof attached to a Waters UPLC (Waters, Cambridge, Massachusetts, United States). The liquid chromatography mobile phases, gradient flow rates, and columns were used as previously published^23,40^. Mass spectra were acquired using negative-mode electrospray ionization run in MSE continuum mode. The metabolite raw signals were quantified by retention time and accurate mass using MassLynx Version 4.2 (Waters). Acetyl-CoA levels were determined by measuring the response (unlabeled acetyl-CoA signal: ^13^C_2_-acetyl-CoA signal) in each sample. Concentration was determined by a 5-point calibration curve containing unlabeled acetyl-CoA (0.005–5 µM) and ^13^C_2_-acetyl-CoA at a constant 2 µM. Since isotopic labeled standards of CoA were unavailable, the standard addition method was used to measure CoA concentrations and correct for matrix effects. Briefly, CoA raw signal was measured in the samples used for acetyl-CoA analysis and in the sample with 1 µM unlabeled CoA standard added. To account for signal carry-over, CoA raw signal was corrected with the average % carry-over. The average % carry-over was determined by averaging % carry-over signal of CoA in the blanks and the sample run on the instrument prior to the blank. For all samples, the raw CoA signal was corrected for carry-over by the following equation: (corrected raw CoA signal) = (raw CoA signal)—(the average % carry-over) X (raw CoA signal in sample run prior on the instrument).

### Clinical chemistry and hepatic acetate quantitation

β-Hydroxybutyrate levels in undiluted serum samples were assessed using a commercially available kit (Sigma-Aldrich) according to manufacturer’s protocol. Similarly, acetate levels were assessed liver samples (~ 40 mg) using a commercially available kit (Sigma-Aldrich) according to manufacturer’s protocol. An Infinite M200 plate reader (Tecan, Durham, North Carolina) was used to assay all replicates.

### Protein extraction and quantification

Frozen liver tissues (~ 50 mg) were homogenized in RIPA buffer with protease inhibitors (Sigma-Aldrich) using a Polytron PT2100 homogenizer (Kinematica, Lucerne, Switzerland) followed by sonication on ice. Samples were centrifuged, after which the supernatant was collected, and protein concentration measured using a bovine serum albumin standard curve and a bicinchoninic acid (BCA) assay (Sigma-Aldrich).

### Capillary electrophoresis protein analysis

The WES capillary electrophoresis system (ProteinSimple, San Jose, CA) was used following standard manufacturer protocols to assess protein levels on total liver lysates. Compass for SW (v4.0.0; ProteinSimple) was used to assess the area under each peak using the Gaussian fit method. The following antibodies and dilutions were used from the respective manufacturers: PDH (1:50; #2784; Cell Signaling, Danvers, MA); PDH p-Ser300 (1:50; AP1064; Sigma-Aldrich); ACLY (1:65; #4332; Cell Signaling); Total Acetylated Lysine (1:65; #9441; Cell Signaling); AMPK (1:50; #2603; Cell Signaling); AMPK p-Thr172 (1:50; #2535; Cell Signaling); ATG14 (1:50; PD026MS; MBL International, Woburn, MA); ATG14 p-Ser29 (1:50; #92340; Cell Signaling).

### Western blotting

Protein samples (20 μg) from total liver lysates were resolved via 10% SDS-PAGE gels (Bio-Rad, San Diego, CA, USA) and transferred to nitrocellulose membranes (GE Healthcare, Chicago, IL) using the Mini Trans-Blot Cell Unit (BioRad) by wet electroblotting (100 V, 45 min). The membranes were then blocked with 5% nonfat milk (in Tris-buffered saline [TBS] + 0.01% Tween) for 1 h and incubated with primary antibodies: anti-β-hydroxybutyryllysine (1:1000; PTM-1201; PTM Biolabs, China) or anti-β-actin (1:1000; #4970; Cell Signaling) overnight at 4 °C. Blots were visualized using horseradish peroxidase (HRP)-linked secondary antibodies (1:3,000; Cell Signaling Technology) and an ECL kit (Millipore Corporation, Billerica, MA). Membranes were scanned on a Sapphire Biomolecular Imager (Azure Biosystem, Dublin, CA). Protein density values were assessed and calculated using ImageJ software (version 1.47; National Institutes of Health, Bethesda, MD). The expression for the protein of interest was standardized to β-actin levels.

### Protein localization data

Protein subcellular localization data were acquired using COMPARTMENTS^95^ as previously described^40^. Subcellular localizations are listed using the following abbreviations unless otherwise noted: cytosol (C), endoplasmic reticulum (ER), extracellular space (ES), Golgi apparatus (GA), lipid droplet (LD), lysosome (L), mitochondrion (M), mitochondrial outer membrane (OMM), mitochondrial inner membrane (IMM), nucleus (N), peroxisome (P), and plasma membrane (PM).

## Supplementary Information

Supplementary Figures.

## Data Availability

Hepatic RNA-seq data sets were previously published^23,41,96^. Differentially expressed genes (|fold-change|≥ 1.5 and posterior probability (P1(t)) ≥ 0.8) were determined by empirical Bayes analysis^97^. Time course (GSE109863), dose response (GSE203302), and diurnal rhythmicity (GSE119780) sequencing data are available at the Gene Expression Omnibus. Diurnal rhythmicity was determined using JTK_CYCLE as previously described^23^. AHR ChIP-seq (GSE97634) and computationally identified putative dioxin response elements (pDREs, https://doi.org/10.7910/DVN/JASCVZ) data were previously published^41^. ChIP-seq analysis used a false discovery rate (FDR) ≤ 0.05. pDREs were considered functional with a matrix similarity score (MSS) ≥ 0.856 and associated with genes when located 10 kb upstream of the transcription start site (TSS) to the transcription end site (TES). Raw metabolomic data were previously published^40^ and can be access through the NIH Metabolomics Workbench (ST001379).
