# Supplementary material for: Consequences of reprogramming acetyl-CoA metabolism by 2,3,7,8-tetrachlorodibenzo-p-dioxin in the mouse liver
Source: Sci Rep. 2023 Mar 13;13:4138. doi: 10.1038/s41598-023-31087-9 (PMC10011583; doi:10.1038/s41598-023-31087-9)
Supplement: Supplementary file 1 — Supplementary Figures. [file 41598_2023_31087_MOESM1_ESM.docx]

**Consequences of reprogramming acetyl-CoA metabolism by 2,3,7,8-tetrachlorodibenzo-*p*-dioxin in the mouse liver**

Giovan N. Cholico^1,2^, Karina Orlowska^1,2^, Russell R. Fling^2,3^, Warren J. Sink^1,2^, Nicholas A. Zacharewski^1^, Kelly A. Fader^1,2^, Rance Nault^1,2^, Tim Zacharewski^1,2,*^

^1^Biochemistry & Molecular Biology, Michigan State University, East Lansing, MI 48824, USA

^2^Institute for Integrative Toxicology, Michigan State University, East Lansing, MI 48824, USA

^3^Microbiology & Molecular Genetics, Michigan State University, East Lansing, MI 48824, USA

^*^Correspondence:

Tim Zacharewski

Michigan State University

Department of Biochemistry & Molecular Biology

Biochemistry Building

603 Wilson Road

East Lansing, MI 48824

tzachare@msu.edu

**SUPPLEMENTARY METHODS**

**Alanine Aminotransferase (ALT) Activity Assay**

Serum was diluted 1:10 in phosphate-buffered saline (PBS). ALT content was measured using the Infinity^TM^ ALT (GPT) Liquid Stable Reagent according to the manufacturer’s protocol (Thermo Fisher Scientific, Waltham, MA). Samples were assessed in technical duplicates and ALT content was expressed as activity in U/L. An Infinite M200 plate reader (Tecan, Durham, North Carolina) was used to assay all replicates.


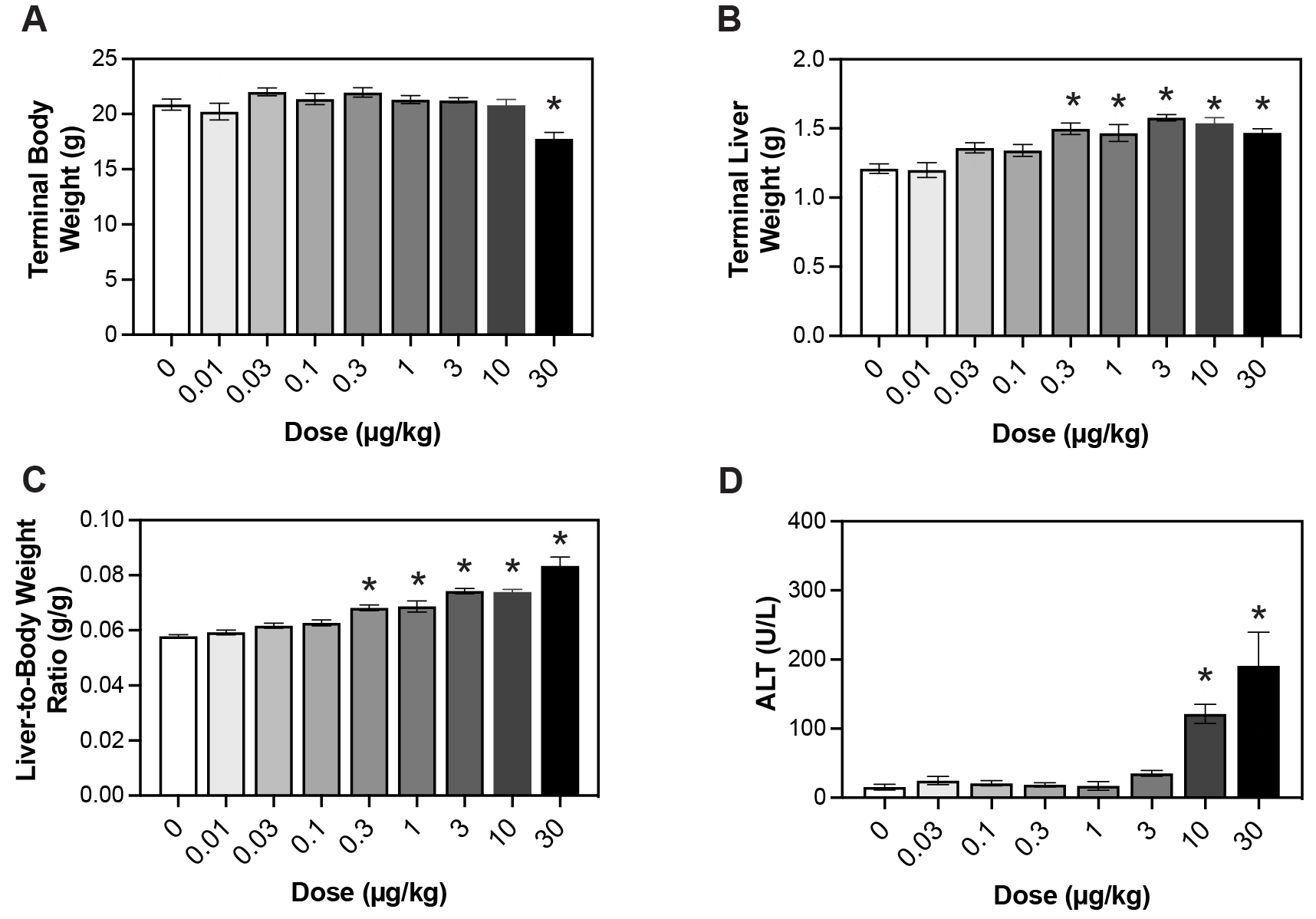


**Supplementary Figure 1:** Mice (n = 9) were orally gavaged every 4 days for 28 days with 0.01, 0.03, 0.1, 0.3, 1, 3, 10 or 30 μg/kg TCDD, or sesame oil vehicle, prior to tissue collection between ZT 0-3. Terminal **(A)** body and **(B)** absolute liver weights were assessed for each treatment group. **(C)** Data were normalized by calculating the liver-to-body weight ratio. **(D)** Hepatotoxicity was assessed using alanine aminotransaminase (ALT) activity in serum (n = 5). Bar graphs denote the mean ± SEM. Significance (*p ≤ 0.05) was determined using a one-way ANOVA followed by Dunnett’s *post-hoc* analysis. Plots were created using GraphPad Prism (v8.4.3).


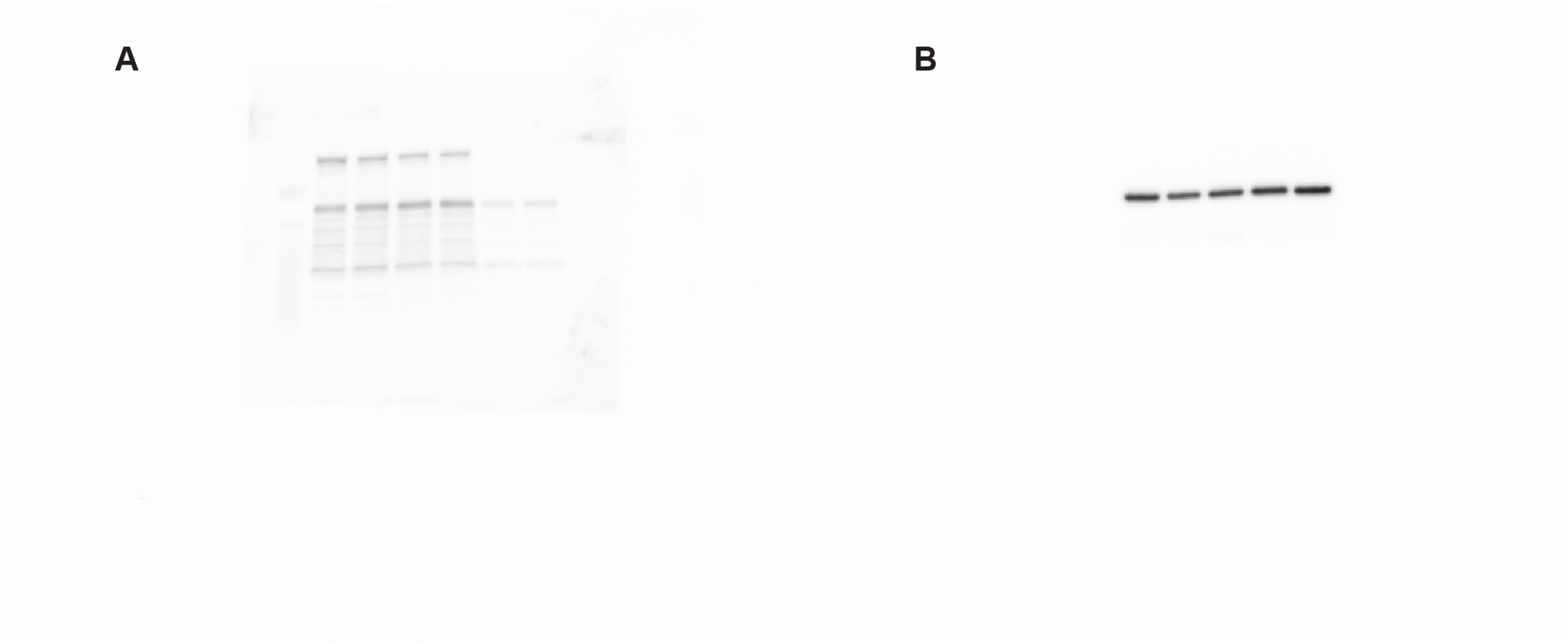


**Supplementary Figure 2:** Western blotting was used to assess total β-hydroxybutyrylated proteins, and normalized to β-actin (n=3). Depicted is a representative raw blot for (**A**) total β-hydroxybutyrylated and (**B**) β-actin. For the total β-hydroxybutyrylated proteins, the lanes from left to right correspond to the ladder, vehicle, 1, 3, 10, and technical duplicates of 30 μg/kg TCDD. For β-actin, the lanes from left to right correspond to the ladder, vehicle, 1, 3, 10, and 30 μg/kg TCDD. The signal intensity of bands at various molecular weights was determined using ImageJ, and are depicted in **Figure 5G**.
